# Wavelet-based information theory in quantitative assessment of AFM images’ quality

**DOI:** 10.1038/s41598-024-53846-y

**Published:** 2024-02-18

**Authors:** Bartosz Czesław Pruchnik, Piotr Adam Putek, Teodor Paweł Gotszalk

**Affiliations:** Department of Nanometrology, Faculty of Electronics, Photonics and Microsystems, Wroclaw University of Science and Technology, 50-372 Wrocław, Poland

**Keywords:** Imaging techniques, Microscopy

## Abstract

The quantitative assessment of the image quality produced by atomic force microscopy (AFM) is an ongoing and challenging task. In our study, we demonstrate Shannon’s application of information theory for measuring image quality. Specifically, we propose quantifying the loss of image information due to the various distortion processes by exploring the relationship between image information based on the information channel capacity (ICC), spectral image representation, and visual quality. Since the ideal image is unavailable, the power and noise spectrum, the critical input information for the image quality evaluation, must be robustly estimated in the proposed method. The classical, most popular Welch method for spectral estimation uses an average of several windowed periodograms and can produce biased spectrum estimates. Therefore, in our work, we discuss an alternative technique based on the wavelet transform that can be applied to solve this challenging problem, specifically in the case of noisy, uncertain AFM measurements. Finally, we validate the performance of the enhanced ICC-wavelet-based algorithm with noisy measurement AFM data.

## Introduction

Scanning probe microscopy (SPM) based technologies, including atomic force microscopy (AFM), have been broadly applied for studying the electrical, mechanical, and other properties of metals, semiconductors, dielectrics, or organic structures. Appropriate signal acquisition can analyse the properties of a sample under consideration. Measurements of force are performed indirectly. The cantilever used in the process is the force-displacement transducer; therefore, measurement is vulnerable to the influence of any external forces. Consequently, uncertainties involved in a measurement process may have various origins. Temperature changes affect the sensor and the sample, changing the noise properties and dimensions. Acoustic noises generated by machines and human activity result in the displacement of the mechanical parts of the detector, influencing the measurement. Electromagnetic noise is constantly present in the background and interferes with the electronic part of the system in the broad spectrum of frequencies. Although several means are undertaken in the system’s design, noises can only partially be sorted out. Therefore, a need emerges to assess the level of noise affecting the measured information quantitatively.

In the past two decades, image quality assessment methods have gained increasing attention from academics and industry for their wide applications in many fields, including compression, fusion, registration, and reconstruction^1^. Concerning used measures, approaches for image quality evaluation can be classified as quantitative and qualitative techniques. These studies focus on the former method, which applies the information-based capacity criterion by Shannon^2^ for image quality assessment in the AFM^3^. More specifically, there are three inspired by information theory approaches to visual quality assessment (QA): (i) with a complete reference model, (ii) with a reduced reference model, (iii) and a no-reference image^4,5^. The availability of reference information has important practical implications in designing the algorithm for the QA of images. This paper deals exclusively with a no-reference quality assessment method for AFM images.

The AFM image quality is metrologically defined as the trueness of the created topographic map to the original surface. There are numerous possible discrepancies, which will be elaborated on in the following sections. More precisely, due to the measurement procedure in a noisy environment, AFM images can suffer numerous artifacts, such as a dust particle stuck to the tip, a drift, the proportional-integral-derivative (PID) loop going haywire, the sudden tip-sample interaction changes during scanning, to stay with a few. As a result, certain factors decrease the signal-to-noise ratio (SNR), while others can keep SNR-like measures unchanged or even enhance them. For instance, on the one hand, the drift might completely ruin measurements of the grating period, but on the other hand, it may be a minor problem for roughness measurement. The precisely opposite effect brings, for example, tip convolution. Besides, a roughness measurement may have significant lateral positioning errors that will not affect the desired measurement result. Moreover, image quality includes imperfections that influence spatial imaging, reducing spatial resolution. Thus, in our work, we focus predominantly on the influence of the Gaussian-like noise on the AFM procedure, mimicking certain aspects of a harsh environment with a Gaussian-like noise model.

The maximum capacity of the information channel (ICC) by Shannon^2^, furthermore generalized in^6^, is explored. According to information theory, the Shannon capacity of a communication channel refers to the maximum amount of error-free information that can theoretically be transferred over the channel without error. From a purely abstract viewpoint, the imaging procedure can be seen as an instance of a communication process. Therefore, assuming that image quality is proportional to the ICC measure, related to the modulation transfer function (MTF) and the level of a perturbed noise^4,5,7,8^, this technique can also be used for image quality evaluation. In particular, Shannon’s metric was also successfully used in^3^ to measure the quality of the AFM imaging process quantitatively. However, this measure might be biased due to the uncertainty of the power spectrum estimator. Thus, choosing the right spectrum analysis tool might be challenging. The state-of-the-art spectrum estimation methods are periodogram^9^, Blackman-Tukey^10^, Welch^11^ and Multi-taper approach^12^. Since the ICC measure might be prone to measurement noise, the wavelet method seems to be a superior tool for spectrum estimation^13^.

On the one hand, this paper’s main contribution is to enhance the assessment method for the quality of the AFM imaging process. On the other hand, an equally important objective is to provide robust spectral estimates from noisy, uncertain AFM measurements, enabling the extraction of a reliable AFM image spectrum. A spectrum estimation approach based on a wavelet transform is proposed for these reasons. The main challenge for wavelets in this application lies in their capability to deal with singularities and irregular structures apart from the trade-offs they offer in terms of varied metrics, including frequency resolution and variance of the estimated power spectrum^14,15^. According to the author’s best knowledge, applying the wavelet transform to quantitative AFM image assessment in the context of information theory has not been studied yet in the proposed framework.

## AFM image quality assessment

In the case of the AFM technique, the signal-to-noise ratio (SNR) plays a crucial role in assessing image quality apart from the MTF. However, calculating those parameters requires measurements on a specially prepared so-called reference sample, which might seem complicated due to manufacturing uncertainties. Moreover, information on the potential improvements in image quality is often demanded in the early stage of the measurement process. For instance, it allows for calibrating the AFM setup. For this purpose, a single parameter methodology was proposed in^3^, which allows for quantitative image quality assessment. Another solution, developed in^16^, relies on estimating the normalized power spectrum’s variance, which requires recording images with relatively high SNR values.

### Information channel capacity as image quality measure

The visual quality quantification process is crucial to various image and video processing applications, including AFM measurements. Within this context, we propose measuring the loss of image information due to the various distortion processes by exploring the relationship between image information based on the information theory by Shannon^17^, its spectral representation, and visual quality. In the following, likewise, as in^4,5,7^, we presume that Shannon’s information capacity could measure perceived image quality determined by the MTF and noise. As a result of this assumption, the imaging procedure is seen in our work as an instance of a communication process. Consequently, Shannon’s information channel capacity (ICC), which is a function of both bandwidth $\mathscr {W}$ and the signal-to-noise ratio $\left( {\frac{{\mathcal{S}}}{{\mathcal{N}}}} \right)$, can be treated as a suitable measure for image quality assessment^3–5,7,16,18^.

According to *Theorem 2*, from^2^, the complete one-dimensional equation for Shannon capacity, derived for the signal perturbed by the white thermal noise of power *N* in the band $\mathscr {W}$, is given by

1 $$\begin{aligned} \mathscr {C} \doteq \mathscr {W}\cdot \log _2 \left( \frac{\mathscr {S}}{\mathscr {N}}+1\right) \end{aligned}$$

with resulting units of information expressed in bits per second, $[\mathrm b/ s]$, where $\mathscr {S}$ denotes the mean power of a signal. The straightforward generalization of Eq. (1) to the case of the arbitrary Gaussian noise, reads as^2,3^

2 $$\begin{aligned} \mathscr {C} = \int _0^\mathscr {W}\log _2 \left[ \frac{{\mathscr {S}}({\xi })+{\mathscr {N}}({\xi })}{{\mathscr {N}}({\xi })}\right] \mathrm{{d}} {\xi }, \end{aligned}$$

where ${\xi }$ denotes the frequency of the spectral component of $\mathscr {S}({\xi })$ and $\mathscr {N}({\xi })$, respectively. More specifically, in Eq. (2), the signal and noise power spectrum under the Gaussian assumption can theoretically be estimated by $\mathscr {P}({\xi }) = \sigma ^2[\mathscr {S}_{\textrm{ideal}}({\xi })]$ and $\mathscr {N}({\xi }) = \sigma ^2[\mathscr {N}({\xi })]$ with variance denoted by $\sigma$, respectively. In particular, we suppose that the measured signal includes noise that results in $\mathscr {P}({\xi })+\mathscr {N}({\xi })=:\sigma ^2[\mathscr {S}({\xi })]$. Finally, we conclude that, as in^2,17^, in our notation $N({\xi })$ denotes the noise measured in the presence of signal $S_{\textrm{ideal}}({\xi })$ (not narrow-band noise of frequency $({\xi })$). Furthermore, it is also worth noting that in Eq. (2), the predominant measure of ICC corresponds to the SNR values, while the bandwidth $\mathscr {W}$ is a result of signal representation only. In addition, the uncertainty of the tested sample design results in uncertainty of its spectrum. Nevertheless, under the assumption that the white model noise is appropriate for the slow scan axis due to the relatively long measurement time, ICC is finally estimated by

3 $$\begin{aligned} \mathscr {C} = \int _0^\mathscr {W}\log _2 \left[ \frac{{\mathscr {S}}({\xi })}{{\mathscr {N}}}+1\right] \mathrm{{d}} {\xi } \approx \int _0^\mathscr {W}\log _2 \left[ \mathscr {F}\left( \frac{{{\mathscr {S}}_{N}}({\xi })}{{\mathscr {N}}}\right) \right] \mathrm{{d}} {\xi }, \end{aligned}$$

where ${{\mathscr {S}}_{N}({\xi })}$ refers to the power spectrum of the noisy signal, and $\mathscr {F}[\,\cdot \,]$ is the function filter responsible for the thresholding operation of values smaller than 1 to avoid integration of negative values of logarithms due to the uncertain power spectrum estimator.

Moreover, one-dimensional Eq. (3) can straightforwardly be adjusted to a 2D case

4 $$\begin{aligned} \mathscr {C}^{2D} \doteq \int _0^\mathscr {W} \int _0^\mathscr {W}\log _2 \left[ \mathscr {F}\left( \frac{{{\mathscr {S}}_{N}}(u,v)}{{\mathscr {N}(u,v)}}\right) \right] \mathrm{{d}} u \mathrm{{d}} v, \end{aligned}$$

where *u* and *v* denote the spectral component frequency in *x* and *y* direction, respectively. Correspondingly, after using a polar coordinate system with the spectral radius denoted by

5 $$\begin{aligned} {\xi }_r = \sqrt{u^2+v^2} \end{aligned}$$

with unit (μ m^−1^) and considering the weak dependence $\mathscr {S}$ and $\mathscr {N}$ on $\theta$, it can finally be transformed into an equation in one dimension as

6 $$\begin{aligned} \mathscr {C}^{1D}_{r} = \int _0^{2\pi } \int _0^\mathscr {W}\log _2 \left[ \mathscr {F}\left( \frac{{{\mathscr {S}}_{N}}({\xi }_r,{\xi }_{\theta })}{{\mathscr {N}({\xi }_r,{\xi }_{\theta })}}\right) \right] \,{\xi }_{r} \mathrm{{d}} {\xi }_{r} \mathrm{{d}} {\xi }_{\theta } \approx \kappa \int _0^\mathscr {W}\log _2 \left[ \mathscr {F}\left( \frac{{{\mathscr {S}}_{N}}({\xi }_r)}{{\mathscr {N}}}\right) \right] \,{\xi }_{r} \mathrm{{d}} {\xi }_{r} \end{aligned}$$

with $\kappa ={2\pi }$ and the constant level of white noise denoted by *N*. The transformation defined by Eq. (6) can also be justified by the fact that significant variation is predominantly concentrated at specific scales in the centre of the 2D power spectrum. Thus, the one-dimensional power spectrum is expressed as

7 $$\begin{aligned} {{\mathscr {S}}^{\textrm{1D}}_N({{\xi }_r})_{m} = \int _{{{\xi }_r}_m}^{{{\xi }_r}_{m}+m\Delta {{\xi }_r}} \mathscr {S}^{\textrm{2D}}_{{N}}\left[ \sqrt{u^2+v^2}\,\right] \; \textrm{d}{\xi }_r, \quad m=1,\ldots ,M.} \end{aligned}$$

where $\mathscr {S}^{\textrm{2D}}_{\mathscr {N}}[\cdot]$ denotes the 2D centred power spectrum, approximated by Eq. (8). The unit of the estimated $\mathscr {C}^{1D}_{r}$ is given in bits per μm (b/μm). It shows the maximum amount of bits of information that (b/μm) of the scanned distance can transmit^3^. The appropriate reduction of the dimensionality through equivalence classes was also recommended by^2,7,17^. There exist varied methods which allow for estimating the power spectrum ${{\mathscr {S}}_{N}}({\xi })$ for example, the periodogram technique^9^, the Blackman-Tukey method^10^, and the Welch approach^11^. Another group of algorithms for the power spectrum estimation is the multitaper spectrum estimator proposed by Thomson^12^ and its natural extension, the wavelet packet approach^13^.

### Fourier transform-based method for power estimation

**Figure 1:**
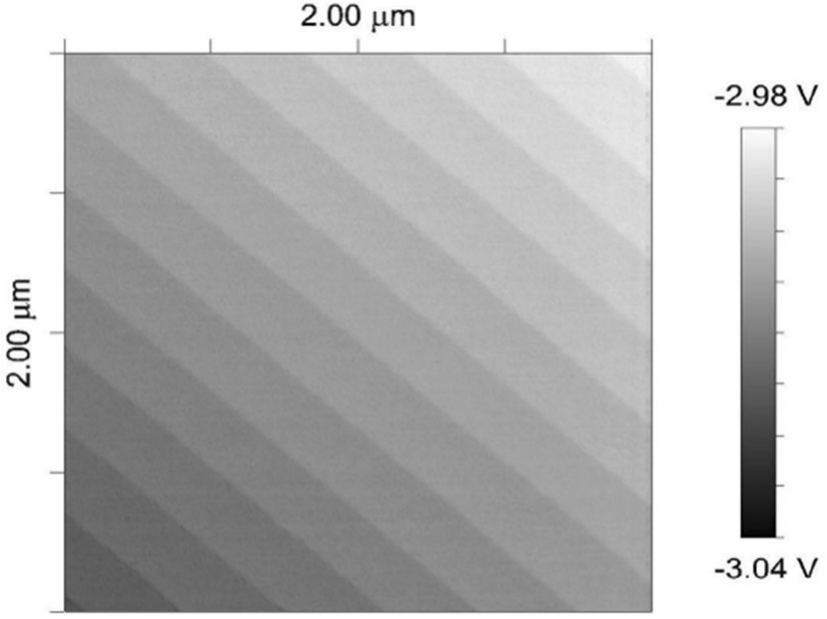
Example of AFM measurement result.

The common feature of the first group method^9–11^ to estimate the power spectrum is the usage of the two-dimensional (2D) Fourier transform. Denote by $g(x, y) \in L^2(\textbf{R}^2)$ a square-integrable function whose domain is the spatial location within an image located at coordinates, (*x*, *y*), and whose gray level intensity range at this location is bounded by $0 \le I_v \le 255$. Here, 0 is related to black, while 255 corresponds to white. The exemplary result of the AFM measurement is depicted in Fig. 1. It is treated in our research as the case study for it is not burdened with purposely generated noise. In the experimental part, samples were measured in three different configurations using various intensities of correlated noise. Only based on them the actual information channel capacities were calculated, contributing to the case study. Out of conducted measurements and calculations, several are presented as appendices to the manuscript.

In the classic approach, measurement data *g*(*x*, *y*) are transformed by a windowed 2D Fourier transform given in the continuous form by^19,20^

8 $$\begin{aligned} {{\mathscr{S}}^{\textrm{2D}}_N(u,v)}=\left( \hat{g} \circ {\hat{h}} \right) \left( u,v;x_1,y_1 \right) \doteq \int _{-\infty }^{\infty }\int _{-\infty }^{\infty } g(x,y) \textrm{e}^{-2\pi j(ux+vy)} h(x-x_1, y-y_1)\textrm{d}x\textrm{d}y\; \end{aligned}$$

with $j=\sqrt{-1}$, $x_1 = nx_0, y_1 = my_0, u = lu_0,$ and $v = pv_0$, $n,m,l,p \in \textbf{N}$, where $\circ$ represents an windowing operator with the window function denoted by *h*(*x*, *y*).

**Figure 2:**
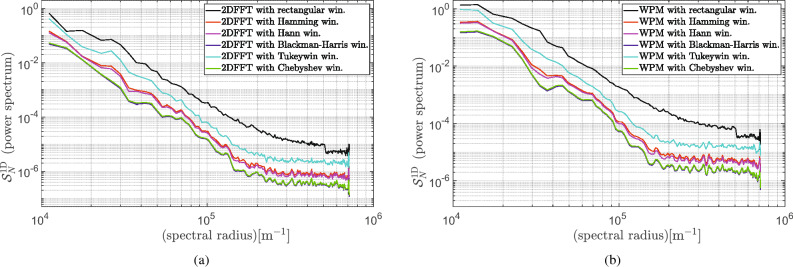
1D power spectrum with varied windows function calculated for the case study sample: (**a**) 2DFFT periodogram; (**b**) Welch periodogram method.

Furthermore, the application of Parseval’s theorem, which asserts that the Fourier transform preserves energy, yields the estimation of the power spectrum

9 $$\begin{aligned} \int _{-\infty }^{\infty }\int _{-\infty }^{\infty } |g(x,y)|^2 {\textrm{d}}x{\textrm{d}}y = \int _{-\infty }^{\infty }\int _{-\infty }^{\infty } | \hat{g} (u,v)|^2 \textrm{d}u\textrm{d}v \end{aligned}$$

with the standard $\ell ^2$-norm (the Euclidean norm) defined by $|\cdot |$. It is a well-known fact that the result of the power estimation depends strongly on the window function to be used^9^. In the simplest form, the periodogram utilizes a rectangular window. However, its counterpart in the frequency domain is a ${\sin}c$ function, which results in a high sidelobe and significant leakages in the power estimates. For this reason, a window function with a taper that smoothly decays on both sides is used^21^, for example, Hamming, Blackman-Harris, Tukey and Blackman. The second serious issue with estimating power spectral density with the periodogram is that it results in significant variance and low precision, which cannot be alleviated using more data. The averaging operation of power spectral density is often used as a remedy. The so-called Blackman-Tukey method and the Welch periodogram originated from this idea. For instance, the 1D power spectrum was calculated using Eq. (7) for both presented methods and shown in Fig. 2a and b, respectively.

**Figure 3:**
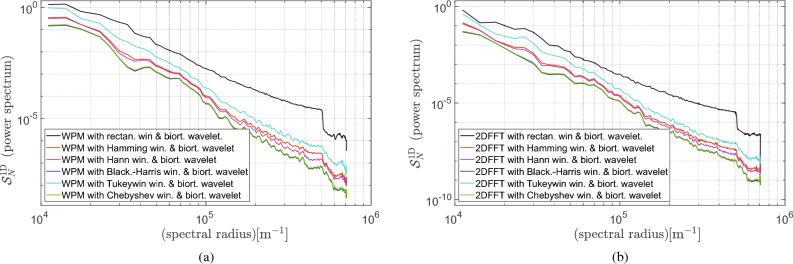
1D power spectrum with varied windows function and additionally denoised with the aid of a biorthogonal wavelet (bior4.4) calculated for the case study sample: (**a**) 2DFFT periodogram; (**b**) Welch periodogram method.

In addition, we also investigated the influence of wavelet denoising properties on the smoothness of the 1D power spectrogram calculated by either the standard 2DFFT periodogram or the Welch periodogram method (WPM). However, since the noise reduction procedure can also remove information while reducing *N*(*f*) at high spatial frequencies below its proper appriori unknown value, and therefore, potentially affecting the MTF (sharpness) of the image, information capacity measurements should be analyzed with caution. Results of filtering 2D AFM image of the case study sample when using the biorthogonal wave function (Specifically, in our computation, (bior4.4) has been used for filtering 2D AFM image which is implemented in MATLAB filter bank.)^23^ are depicted on Fig. 3a and b, respectively. Since a spike on a relatively smooth power spectrum curve can be easily identified, one can find the cutoff frequency and then estimate the noise level as the average value of the flat part of the spectrum^24^. This might, in turn, allow for designing a fully automated algorithm for the quality assessment of AFM images.

### Wavelet-based approach for power estimation

The multitaper spectrum estimator is another solution that improves vorticity and reduces sidelobe and leakage by developing a set of orthogonal tapers or windows^25^. The wavelet-based approach for spectral estimation is the natural extension of that method, which also uses different orthogonal decompositions as prototype filters^14,15^. In the continuous form, a two-dimensional mother wavelet, $\psi (x,y)$ with dilatation and translation controlled by $(a_1, a_2)$ and $(b_1, b_2)$, respectively, is defined as^20^

10 $$\begin{aligned} \psi ^{(a_1, a_2)(b_1,b_2)}\left( x,y\right) \doteq \frac{1}{\sqrt{a_1\cdot a_2}} \psi \left( \frac{x-b_1}{a_1},\frac{y-b_2}{a_2}\right) , \end{aligned}$$

where $a_i,b_i \in \textbf{R}$ and $a_i \ne 0$. Analogously as in the case of the Fourier transform, Parseval’s theorem in the wavelet framework states as

11 $$\int_{{ - \infty }}^{\infty } {\int_{{ - \infty }}^{\infty } g } (x,y)\overline{{\psi (x,y)}} {\mkern 1mu} {\text{d}}x{\text{d}}y = \int_{{ - \infty }}^{\infty } {\hat{g}} (u,v)\overline{{\hat{\psi }(u,v)}} {\mkern 1mu} {\text{d}}u{\text{d}}v$$

with $\overline{u}(x)$ corresponded to a complex conjugate of *u*(*x*), where the Fourier transform of the wavelet function is given as

12 $$\begin{aligned} \hat{\psi }^{(a_1, a_2)(b_1,b_2)}\left( {u,v}\right) =\int _{-\infty }^{\infty }\int _{-\infty }^{\infty } \textrm{e}^{-2\pi j(ux+vy)} \psi \left( \frac{x-b_1}{a_1},\frac{y-b_2}{a_2}\right) \,\textrm{d}x\textrm{d}y.\; \end{aligned}$$

Form Eq. (11) it can be concluded that the wavelet transforms also preserves energy conservation. However, the definition of the wavelet power spectrum as the squared amplitude of the wavelet transform^26^ might be troublesome from a physical perspective due to units. Besides, the power wavelet spectrum calculated this way might be distorted and biased in terms of large scales^27^. A few solutions to this problem are given in^28,29^. In the end, the two-dimensional wavelet transform of *g*(*x*, *y*) is given by^20^

13 $$\begin{aligned} {{\tilde{\mathscr {S}}}^{\textrm{2D}}_N(a,b)}= \left( \hat{g} \circ \psi \right) \left( (a_1, a_2),(b_1,b_2)\right) \doteq \frac{1}{\sqrt{a_1\cdot a_2}}\int _{-\infty }^{\infty }\int _{-\infty }^{\infty } g(x,y)\, \overline{\psi \left( \frac{x-b_1}{a_1},\frac{y-b_2}{a_2}\right) }\,\textrm{d}x\textrm{d}y.\; \end{aligned}$$

In the case of the wavelet transform, calculating a wavelet radius requires only averaging the resulting matrix versus other parameters such as *a* or $\theta$. Many distinct types of wavelets^26^ have found a broad application in the image and signal processing^14,15,23^. In particular, selecting an appropriate wavelet function, $\psi$ is a crucial issue when comparing the radially averaged Fourier power spectrum against the global wavelet power spectrum^30,31^. It results from the fact that this function determines the distribution of the wavelet spectrum. A wide wavelet function provides a more smooth spectrum due to a similarity measure (a scalar product) between each frequency signal component and the windowed wavelet function, which is used for power spectrum estimation. Specifically, the well-known Morlet type wavelet,^27,28^, which employs more sinusoidal cycles than other wavelet transforms to form the analyzing wavelet, seems particularly attractive for this purpose^29,32^. Moreover, since the Morlet wavelet transform is defined in the complex domain, the Morlet power spectrum and the Fourier spectrum can be interpreted similarly. The 2D Morlet complex wavelet (or Gabor wavelet) consists of a complex exponential, which is multiplied by a Gaussian window with associated directional dependence as^33^

14 $$\begin{aligned} \displaystyle \psi _{\textrm{M}}(x,y;\omega _0,\sigma ,\varepsilon , \theta )\doteq e^{\varepsilon j \omega _0\left[ \cos (\theta )x+\sin (\theta )y \right] }\,e^{\frac{1}{2 {\sigma ^2} }\left[ {x^2+y^2}\right] } \end{aligned}$$

with $\sigma \in \mathbb {R}$, $\omega _0 \in \mathbb {R}$, $\varepsilon \in \mathbb {R}$. To fulfill the admissibility condition $\omega _0 = 5.336$^31^.

**Figure 4:**
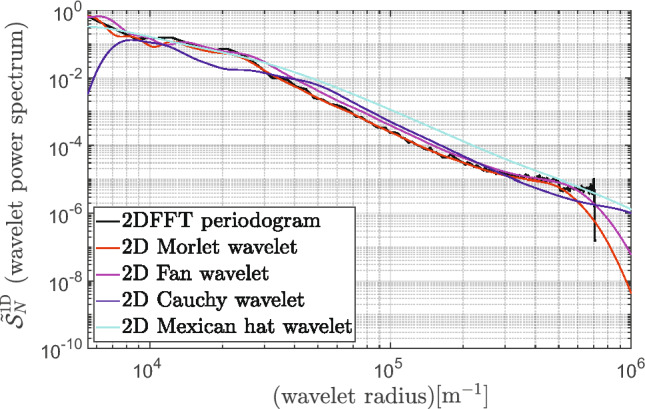
Wavelet spectrum compared to standard 2DFFT periodogram.

Another type of wavelet used for a power spectrum analysis is a Fan wavelet function, which is, in essence, a summation of several directional 2D Morlet wavelets spaced along with different directions to have a direction insensitive amplitude response^34^. Thus, the superposition of the Morlet wavelet is average^32^

15 $$\begin{aligned} \displaystyle \psi _{\textrm{F}}(x,y;\sigma ,\varepsilon , N)\doteq \frac{1}{N}\sum _i^N e^{\varepsilon j \omega _0\left[ \cos (\theta _i)x+\sin (\theta _i)y \right] }\,e^{\frac{1}{2 {\sigma ^2} }\left[ {x^2 +y^3}\right] } \end{aligned}$$

over a finite number of directions with $\theta _i = \theta _0+i\delta \theta$, where $\delta \theta =16.29^{\circ}$ for $\omega _0 = \pi \sqrt{2/ \ln 2} \approx 5.336$. In work^32^ reported that the Fan and Morlet wavelets belong to the most successful in reproducing the Fourier power spectra. It can be explained by pointing out that these wavelets are, in the space domain, complex exponentials modulated by a Gaussian envelope, which are, in fact, very similar to the Fourier basis functions. For instance, the wavelet-based spectrum using varied mother wavelet function is compared to the classic 2DFFT periodogram technique in Fig. 4.

The following section uses Fourier and wavelet transform for 1D spectrum estimation of AFM images.

## Numerical results and discussion

The proposed wavelet-based ICC method for AFM quality assessment was verified using three data series , which include an academic benchmark with generated noised synthetic data, and the analyzed referenced sample affected by two type of various noise sources.

### Benchmark with synthetic white noise

**Figure 5:**
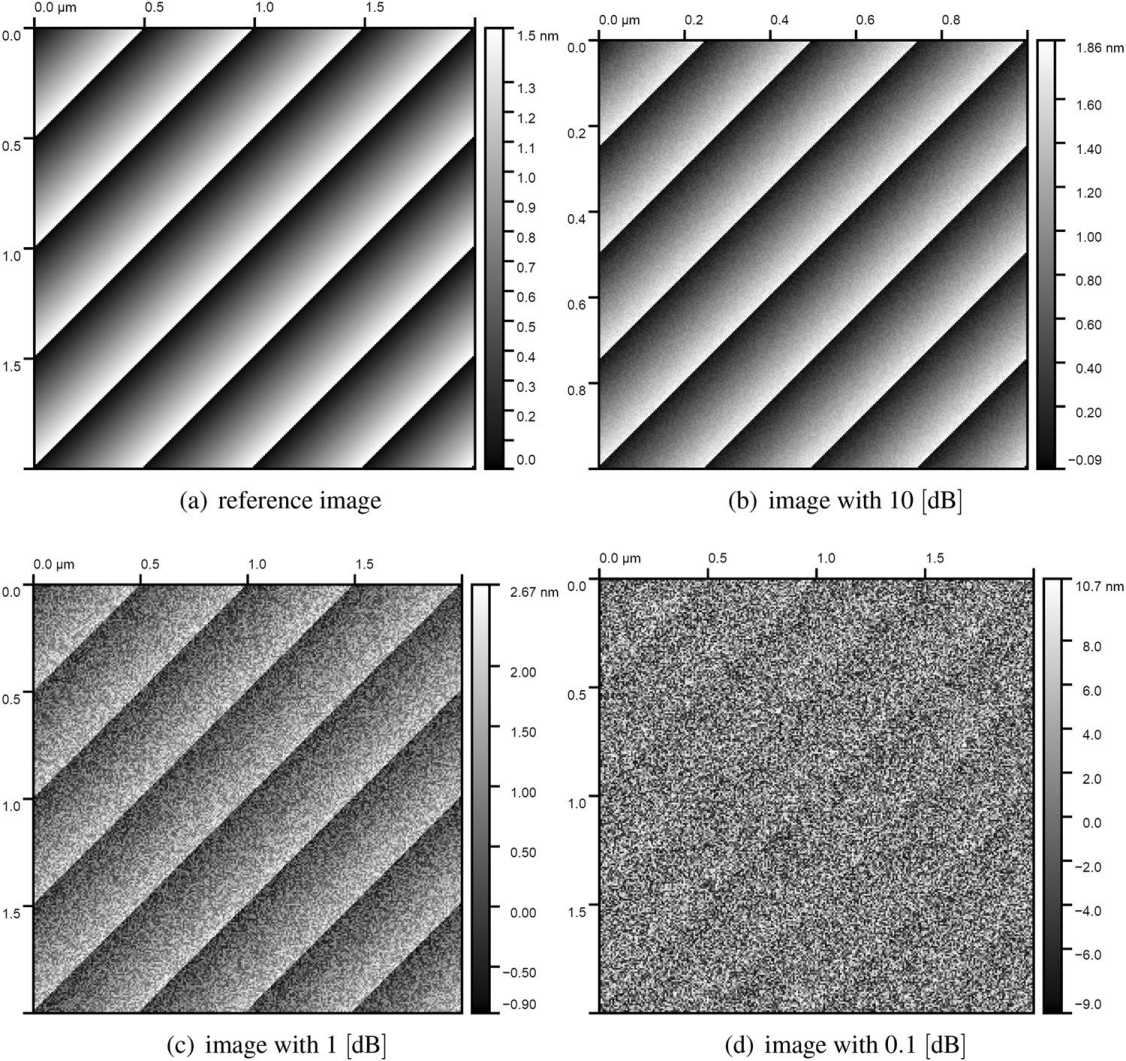
Image generated artificially with various level of synthetic white noise in the range of 10, 1 and 0.1 dB.

To validate the procedure for assessing image quality based on ICC metrics compound with various spectral analyses, we generated artificially an image, shown in Fig. 5, which served as the academic test case. The artificial surface described with the sawtooth signal profile was generated because of a resemblance to the actual measured sample. It was further burdened with white noise generated by a pseudo-random numbers generator recalling the values from a tabularized Gaussian distribution. The matrix of resulting noise was normalized to values of signal-to-noise ratio in the range of 10, 1 and 0.1 dB and subsequently summed with corresponding values of the surface model.

Furthermore, we applied the formula (3) with the 2DFFT periodogram with $i,j=0,\ldots , P$, $P=255$, Welch periodogram, and wavelet-based method to estimate the ICC metric. As recommended in^27,29,32^, we have used the Morlet and Fan wavelets $\psi _{\textrm{M}}(6,1,1)$ and $\psi _{\textrm{F}}(6,1,1,8)$ to calculate the wavelet-based power spectrograms, defined by (13) with (14) or (15). For this reason, following^26^, we have specified the simulation setup: $s = s_02^{j/J}= [1,\ldots ,180.76]$ with the smallest resolvable scale given by $s_0=1.0144$ and an integer $j=[0,\ldots ,J]$ corresponding to the level number with $J=363$, and, finally, a uniform grid for $\theta = l\pi /L = [0,\ldots ,3.115]$ with $l=0,\ldots , L$, $L = 63$.

Furthermore, to approximate the ICC measure (7), we used the classic trapezoidal method with $N+1$ evenly spaced points^22,35^

16 $$\begin{aligned} \mathscr {S} \doteq \kappa \int _{\xi _a}^{\xi _b} f(\xi ) \text{ d } \xi = \kappa \,\xi _h\sum _{n=1}^{N}\left( f(\xi _n)+f(\xi _{n+1}) \right) , \qquad \xi _h = \frac{\xi _b-\xi _a}{N}, \end{aligned}$$

with a posteriori error estimation given by

17 $$\begin{aligned} \delta = \eta f''(\xi )(\xi _b-\xi _a)\xi _h^2 \le |\eta | (\xi _b-\xi _a)\xi _h^2 \big | \sup _{\xi _a \le \xi \le \xi _a} f''(\xi )\big | \approx |\eta | \frac{\left( \xi _b - \xi _a\right) ^2}{N^2}\left[ f'(\xi _b) - f'(\xi _a) \right] , \qquad \xi \in [\xi _a, \xi _b], \end{aligned}$$

where for the last term of (17), an asymptotic error estimate for $N\rightarrow 0$ is conducted with $\eta =-\pi /6$ and the first and second derivative denoted by $f', f''$, respectively. Please note that the approximation error is proportional to $\xi _h^2$ and depends on the regularity of spectrum given by $f(\xi )$.

However, if a (spectrum) function $f(\xi )$ has no bounded a second derivative, i.e. is only a ’rough’ continuous function, the error bound given by (17) is not applicable any more. In such a case, the following sharp error bound for the trapezoidal rule has to be applied^36^

18 $$\begin{aligned} \tilde{\delta } \le \kappa \frac{\left( \xi _b-\xi _a\right) ^2}{8N}\left[ \sup _{\xi _a \le \xi \le \xi _a} f'(\xi ) - \inf _{\xi _a \le \xi \le \xi _a}f'(\xi ) \right] , \end{aligned}$$

where $\sup$ and $\inf$ denotes suppremum and infimum over a set *I*, respectively.

In fact, since the spectral analysis has been applied to the sawtooth signal profile, we suppose that the sharp error bound given by (18) is the reliable error estimation. Finally, the results with error analysis provided by (18) estimate are listened in Table 1. In summary, we can conclude that based on the provided error analysis, the ICC measure wavelet approximation seems less biased than those estimations provided by Welch and 2FFT periodogram method, at least for a moderate, practically occurred noise levels. It is strictly related to the regularity of the integrand and the grid size used for the integration.

**Table 1:** ICC-based metric for artificially generated image with varied white noise level from 10 dB till 0.1dB.

Images shown in Fig. 5	$\textrm{ICC}_{\textrm{2DFFT}}$	$\frac{\tilde{\delta }_{\textrm{2DFFT}}}{ICC_{\textrm{2DFFT}}}$	$\textrm{ICC}_{\textrm{WPM}}$	$\frac{\tilde{\delta }_{\textrm{WMP}}}{ICC_{\textrm{WMP}}}$	$\textrm{ICC}_{\psi _{\textrm{M}}}$	$\frac{\tilde{\delta }_{\psi _{\textrm{M}}}}{ICC_{\psi _{\textrm{M}}}}$	$\textrm{ICC}_{\psi _{\textrm{F}}}$	$\frac{\tilde{\delta }_{\psi _{\textrm{F}}}}{ICC_{\psi _{\textrm{F}}}}$
Unit	(b/μm)	(%)	(b/μm)	(b/μm)	(b/μm)	(%)	(b/μm)	(%)
Pure image (reference)	3.18	0.41	3.20	0.34	3.84	0.26	3.95	0.25
Image with 10 dB	2.94	0.44	2.95	0.37	3.56	0.28	3.67	0.27
Image with 1 dB	1.95	0.62	1.99	0.48	2.77	0.35	2.93	0.32
Image with 0.1 dB	0.38	1.4	0.69	0.62	0.86	0.53	0.60	0.62

The artificially generated test case allows us to demonstrate the applicability of the proposed approach in the more challenging measurement setting.

### AFM measurements

The first part of the measurement was performed with a white noise signal, in which the root mean square (RMS) value was changed from 0 to 20 V with 5 V step. In the second part, acoustic noise was delivered to the system. The varied music tracks were played during the measurement, acting as the known noise source.

All measurements were performed in contact mode with a sinusoidal speed profile. The speed was one line per second. The scanned area of samples was specified either as 2 by 2 (μm) or 1 by 1 (μm). The reason for the change in sample area was to alter the frequency of details in the topogram. Therefore, the results consisted of a series of AFM images with different noise levels.

### Noise modelling

In our paper, two types of noise inputs were utilized to test the influence of types of external noise sources. A distinction is made between white noise and nondeterministic noise. Electronic processing of the signal burdens it with wideband noises. Those wide spectral signals are often referred to as noises of different colours due to similarities to the light spectrum. Among them is white noise, which characterizes the equal distribution of spectral density over all spectrums. This noise was used to elevate the background noise level in the spectrum homogeneously. Moreover, we applied a generator embedded in the function generator to generate white noise. The noise was generated with different RMS values: 5, 10, 15, and 20 V. The results are to be consulted in the appendix Fig. 1.

Music samples were used to simulate natural noise background in the environment with people. The main concern of this measurement was not necessarily a quantitative assessment of the noise level but the ability to differentiate topograms influenced by different types of music, representing different types of background noises. For that purpose, a few pieces of music were used. They were varied from classical music: “*Étude Op. 10, No. 12*” by F. Chopin, “*Piano Concerto no. 4*” by S. Rachmaninoff, “*Symphony No.9* by A. Dvorak, “*Ride of the Valkyries*” by R. Wagner, to the modern rock and heavy metal: “*You Can’t Teach An Old Dog New Tricks*” by Seasick Steve, “*Stargazers*” by Nightwish, and “*Chippin’ in* by Samurai. Indeed, different music sounds should contribute differently to the overall noise spectrum. The effect of AFM measurement are to be acquainted with in the supplementary material.

The influence of narrowband noises, which come from mechanical sources, especially motor drives present in pumps, fans, and generators - devices characterized by constant engine speed - is not considered in our paper.

### Quantitative assessment of AFM images’ quality

The AFM images of the reference sample were measured to demonstrate the performance of the proposed quality assessment method. These reference samples’ topographies look similar in various configurations of the noise excitation. However, differences in AFM images might be rather identified and assessed qualitatively. A power spectral analysis of images may support the rough observations. For this reason, the ICC-based measure, whose calculation procedure was described in Section "Information channel capacity as image quality measure", was used to assess the quality of the recorded AFM images. The methods for the power spectra estimation, including the 2DFFT periodogram, the WPM, and the wavelet-based periodogram technique, described in Section "AFM image quality assessment", were implemented in Matlab^®^^22^. The implementation of the ordinary periodogram method with the use of the 2DFFT routine and varied windows function has been straightforward.

**Figure 6:**
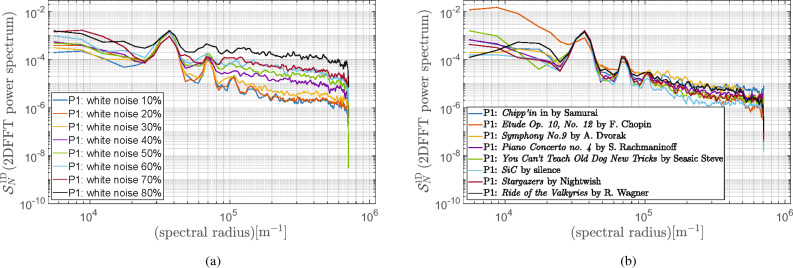
Radial power spectrogram from calculated 2DFFT of reference sample using Blackman-Harris window for: (**a**) white noise; (**b**) music as noise model.

The WPM provides the power spectral density estimate with the aid of overlapped segment averaging estimator, where each segment can be windowed with various types of windows, as stated in Section Fourier transform-based method for power estimation. In our simulations, we considered the maximal size of the segment of 256 by 256 with 50$\%$ overlap. Furthermore, for mapping the resulting 2D power spectra into the 1D space, we used Eq. (7). In the end, the 1D power spectral density plots were prepared for all gathered AFM images with the various source of noise as described in Section Noise modelling. Furthermore, since the spectral analysis provided by the standard 2DFFT periodogram and the WPM look similar, specifically compared to the wavelet-based spectra, only the results for the 2DFFT power spectrum are presented in Fig. 6a and b, respectively.

**Figure 7:**
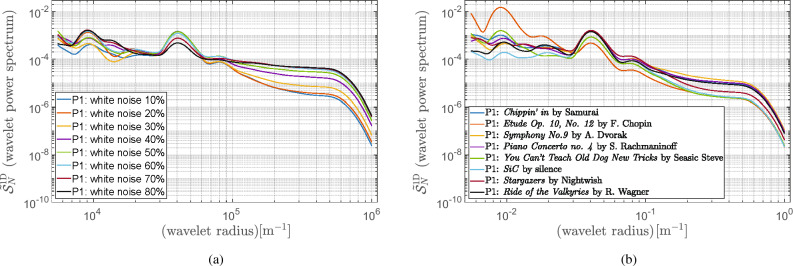
Wavelet power spectrum calculated for reference sample using Morlet wavelet $\psi _{\textrm{M}}(6,1,1)$ for: (**a**) white noise; (**b**) music as noise model.

As to the wavelet-based power spectrograms, we applied the Morlet and Fan wavelets as recommended in^32^. In our simulations, the fast Morlet and Fan wavelet transform^27,29^ was used for wavelets $\psi _{\textrm{M}}(6,1,1)$ and $\psi _{\textrm{F}}(6,1,1,8)$. For convenience, we defined scale *s* as base 2 exponentials^26^, that is, $s = s_02^{j/J}= [1,\ldots ,180.76]$, $s_0=1.0144$ denoted the smallest resolvable scale and an integer $j=[0,\ldots ,J]$ representing the level number with $J=363$. However, we used a uniform grid for $\theta = l\pi /L = [0,\ldots ,3.115]$ with $l=0,\ldots , L$, $L = 63$. To demonstrate the improvements of the wavelet-based method, we compare the 1D wavelet power spectrum results shown in Fig. 7a and b with those provided with the 2DFFT method (cf. Fig. 6a and b) using noised AFM images. It should be noted that the resulting wavelet power spectra’ smoothness properties, on the one hand, allow for automatizing the ICC-based metric of AFM images. For example, a simple procedure for finding an inflection point can be used to identify the background noise. On the other hand, the wavelet-based ICC estimation quality can be significantly improved since the cutoff frequency can be precisely found. In our work, similarly to^24^, the noise level was estimated as the mean value of the flat part of the spectrum $S_{\textrm{N}}(\xi )$. Finally, we explored the ICC metrics proposed in^24^, defined by Eq. (6) with $\kappa =2\pi$ to assess the quality of the recorded AFM images quantitatively. The ICC-based metrics calculated for all gathered AFM images are listed in Tables 2 and 3, respectively.

Adopting the Shannon theorem from the telecommunication technologies^2^, the higher values of ICC will specify the higher quality image. This conclusion results from the fact that the amount of error-free information that may be transmitted through the channel is determined by the bandwidth of the communication channel and the signal-to-noise ratio. In our work, we mainly focused on studying the influence of the signal-to-noise ratio as the limitation for the amount of error-free information. In fact, even a cursory examination of Table 2 allows for verifying this theorem in the context of spectral analysis of AFM images: a higher level of noise limits the quality of images. Even though the ICC metrics computed based on three studied methods for power spectra estimation are consistent, the less biased ones seems to be those provided by the wavelet-based method. It results from the fact that background noise can be identified more precisely when considering a smooth continuous spectrum, which might improve the calculation reliability based on the formula (3).

Furthermore, the ICC measure can also be applied to a noise model, which is random but not equally distributed in the frequency spectrum noise model (cf. Fig. 7a and b). An example of such a noise is music, which was used to simulate a noise produced by a human. Undoubtedly, music, being not perfectly described ambient noise source (especially considering its spectrum), is more of a qualitative indicator and a means to provide noise-differentiated samples into exemplary calculations. Also, in this case, the resulting ICC values summarized in Table 3 produced by three different methods show convergence.

**Table 2:** ICC-based metric for AFM images with varied white noise level 10%−80%.

AFM images	$\textrm{ICC}_{\textrm{2DFFT}}$	$\textrm{ICC}_{\textrm{WPM}}$	$\textrm{ICC}_{\psi _{\textrm{M}}}$	$\textrm{ICC}_{\psi _{\textrm{F}}}$
Unit	(b/μm)	(b/μm)	(b/μm)	(b/μm)
Sample with $10\%$	4.95	4.67	5.49	5.58
Sample with $20\%$	4.32	3.75	5.02	5.14
Sample with $30\%$	3.55	3.25	4.19	4.17
Sample with $40\%$	2.96	2.94	3.56	3.41
Sample with $50\%$	2.58	2.68	3.03	2.84
Sample with $60\%$	2.38	2.54	3.02	2.73
Sample with $70\%$	2.23	2.47	2.63	2.33
Sample with $80\%$	1.91	2.21	2.43	2.01

**Table 3:** ICC-based metric for AFM images noised by varied music types.

AFM images	$\textrm{ICC}_{\textrm{2DFFT}}$	$\textrm{ICC}_{\textrm{WPM}}$	$\textrm{ICC}_{\psi _{\textrm{M}}}$	$\textrm{ICC}_{\psi _{\textrm{F}}}$
Unit	(b/μm)	(b/μm)	(b/μm)	(b/μm)
Sample with *Chippin’*	4.20	3.92	4.31	4.34
Sample with *Etude*	5.51	5.27	5.30	5.33
Sample with *Symphony*	4.67	4.23	4.26	4.15
Sample with *Piano concerto*	4.43	3.69	4.09	4.04
Sample with *Seasick Steve*	5.19	4.56	5.39	5.56
Sample with *SiC*	6.04	5.00	5.54	5.75
Sample with *Stargazers*	5.22	4.90	5.46	5.67
Sample with *Valkyries*	4.41	3.71	4.37	4.16

## Experimental procedures and methods

### Principles of AFM operation and sample characterization

SPM techniques, including electrostatic force microscopy (EFM) or AFM, have found broad application in analyzing the surface properties of varied materials^3,19^. AFM belongs to the SPM family, whose main principle of the measurement is detection of a singular interaction between the probe and the surface - so called atomic forces. Microcantilevers are most often used as probes. In contact mode, a change in sample height is detected by bending a probe by imposing a constant force. Typically, in non-contact mode, a change in the amplitude or frequency of the cantilever vibration is sought, which does not cause surface wear. Additionally, there is no lateral contact force between the probe and the surface, so it is possible to investigate more fragile surfaces (e.g., biofilms, proteins) in this mode^37^. Among the AFM modes, there is also a place for mixed techniques, which combine properties of both contact and non-contact, e.g., a tapping mode in which the probe touches the surface only at specific points, see, e.g., for details^38–42^.

### Experimental set-up

**Figure 8:**
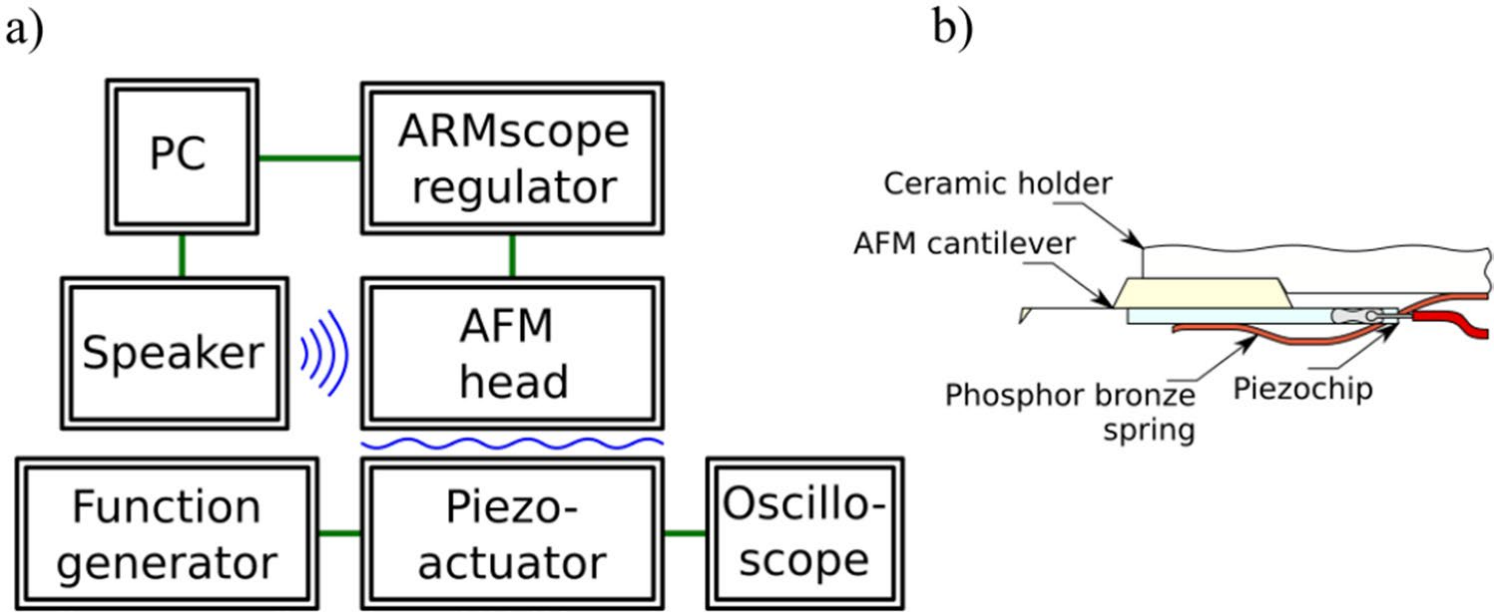
Measuring setup schematics: (**a**) block diagram of used devices and transmitted signals; (**b**) cross-section through a cantilever holder with the included piezoelectric chip (not to scale).

In our studies, we applied the AFM system for contact measurements with an optical beam deflection (OBD) in the experiment. The purpose of conducting measurements was to register signals with different noise levels. A typical AFM system registers low-band signals - in this case, the upper band limit is 10 kHz, therefore the actuator should have a band broader than from 0.1 to 10 kHz and be applied to the cantilever as closely as possible. Both demands are satisfied by a piezoelectric chip actuator. In our research, we used the Thorlabs piezoelectric chip PA4HEW. A phosphor bronze spring held it together with the cantilever in the holder. Schematics of the setup are presented in Fig. 8. The signal to the piezoelectric chip was delivered from the signal generator Tektronix AFG3021B via the infinite impulse response (IIR) low-pass filter with a cutoff frequency 10 kHz. The filter was necessary to eliminate a resonance of the piezoelectric chip (165 kHz).

**Figure 9:**
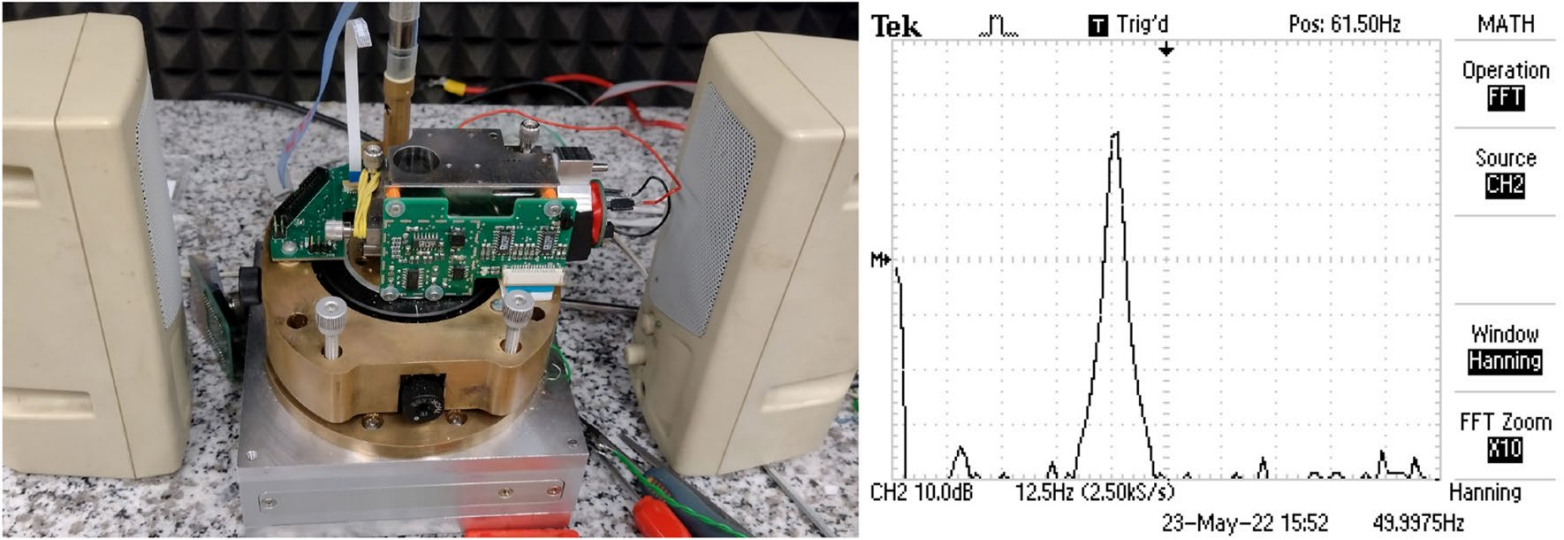
Measuring setup with a source of synthetic acoustic noise and frequency spectrum as measured on cantilever deflection signal for continuous 50 Hz acoustic noise.

In the further phase of our research, we slightly modified the setup to assess the impact of typical acoustic noises on the measurement process. Even though, music usage may seem nonintuitive, we have found it a useful noise source for several reasons. Specifically, we considered music the least consistent noise source, being chaotic in some sense while remaining in a typical frequency range for human activity; concurrently, it is repeatable and of somehow controlled characteristics. The choice of different pieces from different music genres pushed randomization even further, excluding the domination of one set of frequencies. Therefore, it states the intermediate step between a deterministic noise source and an entirely stochastic model. In our case, during the measurements, a noise was emitted with speakers ZX-SPACE ZoltriXound connected to the PC shown in Fig. 9. Speakers were chosen to simulate a noise background typical for the presence of people in the surroundings. They were to emit noise in the acoustic subspectrum between 20 and 10 kHz.

We analysed the same surface in all measurements, SiC with 1.5 nm atomic steps calibration sample. The used cantilevers was a model PPP-CONTSCPt by NANOSENSORS™. They belong to the family of typical contact probes with resonant frequency in the range 1–57 kHz (typically 25 kHz), force constant between 0.01 and 1.87 N/m (typically 0.2 N/m) and full PtIr coating.

The measurement setup offers a sensitivity of 125 nm/V, derived from calibration sample measurements. However, the information capacity of a scanned surface is invariant concerning the transformation of measurements from the voltage to the height domain. In fact, we could even operate on unaltered values as given by the measurement setup.

Measurements performed with the AFM at lower resolutions are highly vulnerable to external noises, which can be present in different forms. In all electronic systems, electrical noises are always present^2,3,24^, yet, the progress in the field of integrated circuits decreases the influence of those noises. Another source is the construction of actuators for height compensation. In dedicated systems, noises are well-characterized and relatively low. The laser detector light is a typical noise source, apart from the mechanical construction (inert elements, material creep) or temperature (thermal expansion, parasitic thermal deflection). External mechanical noises of acoustic and sub-acoustic frequencies seem most influential. Despite active and passive methods of mechanical noise damping, those signals are frequently observed in measurements.

Acoustic noises consist of harmonic and impulse signals, whereby harmonious signals can be filtered out entirely with active and partly with passive damping. Active vibration cancelling relies on generating signals identical to noise in a reversed phase to cancel both signals in superposition. The passive damping is acoustic filtering, so the system’s physical properties limit the filtered band. Another purpose of passive filtering is, on the one hand, to shift the system’s resonant frequency as low as possible to eliminate the possibility of resonant excitation. On the other hand, impulses are easily damped by passive filtering and are impossible to eliminate with active filtration. Nevertheless, harmonious and impulse noises are finally present in the resulting picture.

In the frequency spectrum, noises are visible as peaks corresponding to specific frequencies (mainly harmonious noises), constant band elevation (thermal, electric, and mechanical noises, also impulses), and frequency-dependent band elevation (e.g., 1/*f* noise).

## Conclusion

Our work has briefly explained the methodology of measurements conducted with the AFM setup to address the important problem of the QA process of AFM images, which must be quantitatively assessed to improve the measurement process or find measurements with the highest quality. However, the ideally conducted data series in the sense of the reference measurements do not exist in this case. Therefore, we have had to apply the ICC metric, adopted from telecommunication technologies and initially proposed by^24^. In fact, this metric has been widely used in various applications to assess image quality under the assumption to be correlated with MTF and noise^4,5,7,8^. As this measure is predominantly susceptible to signal-to-noise ratio and image resolution, the superior tool for estimating power spectra is the taper wavelet-based method. On the one hand, it allows for estimating background noise that is a source of bias in the ICC metric. On the other hand, it provides a smooth approximation of power spectra, limiting the spectrum distribution uncertainty. For these reasons, the fast Morlet and Fan wavelet methods have been used in our studies. We used data noised by white noise and a random but not equally distributed frequency spectrum noise model for numerical experiments. Thus, based on our numerical experimentation, we claim that the ICC metric, primarily based on the wavelet approach, could be a robust method enabling the metrological assessment of the recorded AFM images^43^. In addition, applying the wavelet-based method for the power spectrum estimation might result in a fully automatic procedure for AFM image assessment.

The wavelet transform can also be perceived as an intermediate step between filtration and spectral analysis, which allows for extracting essential features of AFM images. It is seen as a promising direction for future investigations.

### Supplementary Information

Supplementary Information.

## Data Availability

The datasets used and/or analysed during the current study available from the corresponding author on reasonable request.
