# Supplementary material for: Wavelet-based information theory in quantitative assessment of AFM images’ quality
Source: Sci Rep. 2024 Feb 18;14:3996. doi: 10.1038/s41598-024-53846-y (PMC10874965; doi:10.1038/s41598-024-53846-y)
Supplement: Supplementary file 1 — Supplementary Information. [file 41598_2024_53846_MOESM1_ESM.pdf]

Appendices

The AFM images of the reference sample with various noise sources are shown in Fig. 1 and Fig. 2

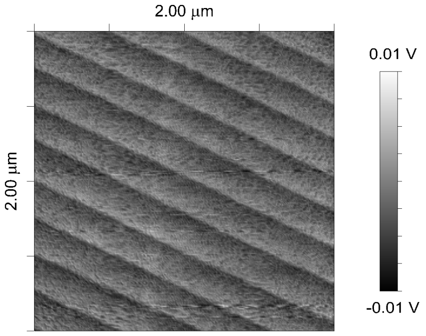

(a) $ICC_{10\%}$: (4.95/4.67/5.49) [b/μm]

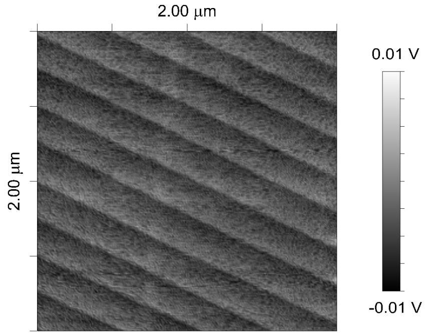

(b) $ICC_{20\%}$: (4.32/3.75/5.02) [b/μm]

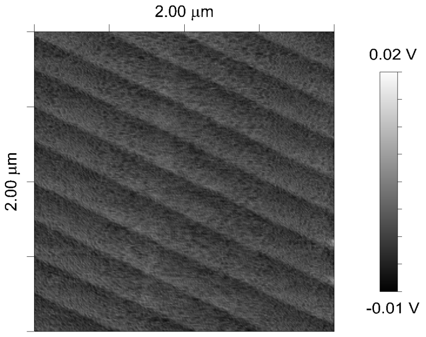

(c) $ICC_{30\%}$: (3.55/3.25/4.19) [b/μm]

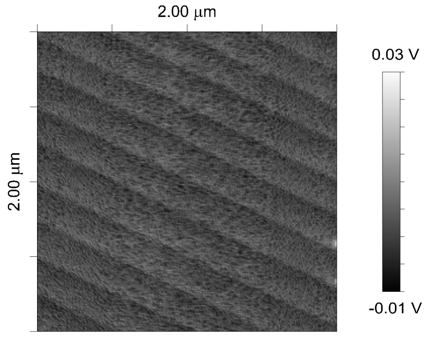

(d) $ICC_{40\%}$: (3.55/3.25/4.19) [b/μm]

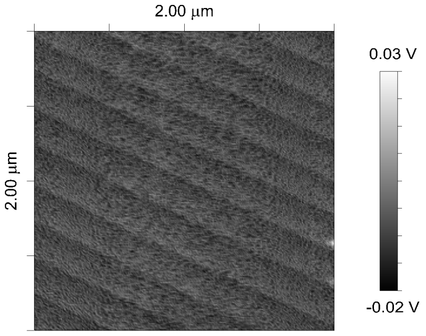

(e) $ICC_{50\%}$: (2.58/2.68/3.03) [b/μm]

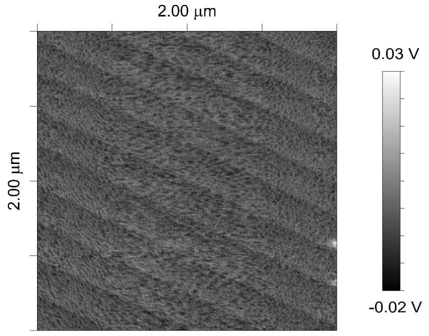

(f) $ICC_{60\%}$: (2.38/2.54/3.02) [b/μm]

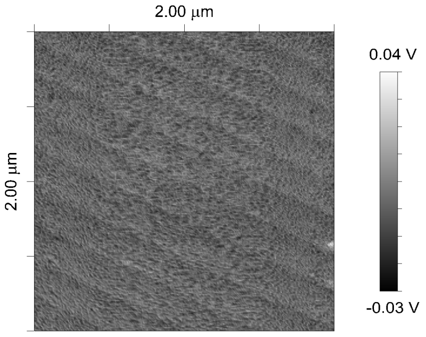

(g) $ICC_{70\%}$: (2.23/2.47/2.63) [b/μm]

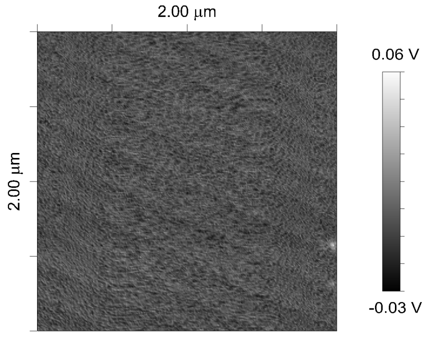

(h) $ICC_{80\%}$: (1.91/2.21/2.43) [b/μm]

Figure 1. ICC metric of AFM images's quality as (ICC_{2DFFT}, ICC_{Welch}, ICC_{ψ_M})[b/μm] for reference sample with various level of white noise.

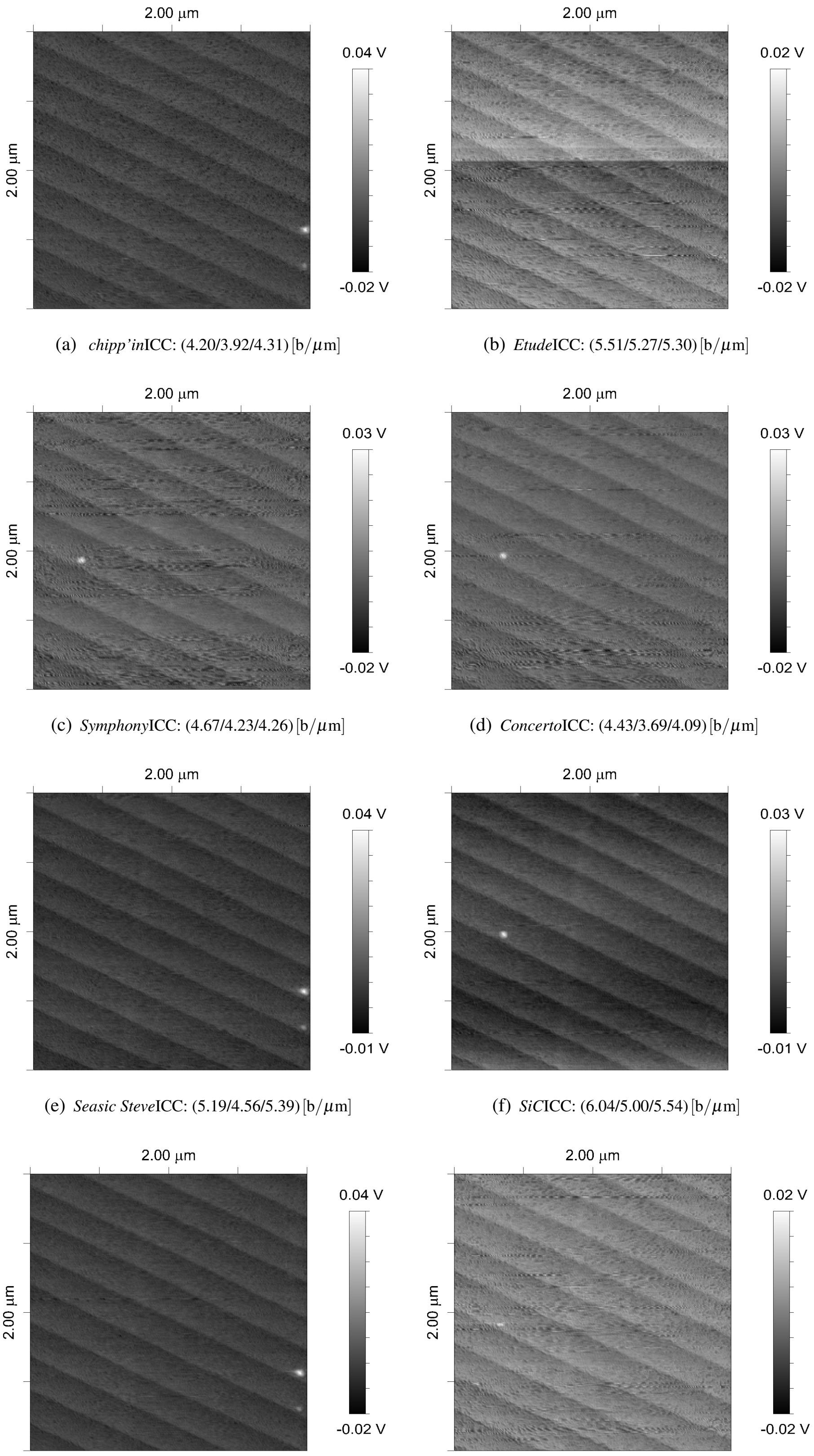

(a) *chipp'in*ICC: (4.20/3.92/4.31) [b/μm]

(b) *Etude*ICC: (5.51/5.27/5.30) [b/μm]

(c) *Symphony*ICC: (4.67/4.23/4.26) [b/μm]

(d) *Concerto*ICC: (4.43/3.69/4.09) [b/μm]

(e) *Seasic Steve*ICC: (5.19/4.56/5.39) [b/μm]

(f) *SiC*ICC: (6.04/5.00/5.54) [b/μm]

(g) *Stargazers*ICC: (5.22/4.90/5.46) [b/μm]

(h) *Valkyries*ICC: (4.41/3.71/4.37) [b/μm]

Figure 2. ICC metric of AFM images's quality as (ICC_{2DFFT}, ICC_{Welch}, ICC_{ψ_M})[b/μm] for reference sample with music as a noise source.
